# Seafood label quality and mislabelling rates hamper consumer choices for sustainability in Australia

**DOI:** 10.1038/s41598-023-37066-4

**Published:** 2023-08-03

**Authors:** Megan E. Cundy, Julia Santana-Garcon, Alexander G. McLennan, Marcelle E. Ayad, Philipp E. Bayer, Madalyn Cooper, Shannon Corrigan, Emily Harrison, Chris Wilcox

**Affiliations:** 1 Flourishing Oceans Initiative, Minderoo Foundation, Perth, WA Australia; 2 Centre for Marine Socioecology, University of Tasmania, Hobart, TAS Australia

**Keywords:** Marine biology, Environmental impact, Conservation biology, Genomics, Sequencing, Sustainability

## Abstract

Seafood mislabelling and species substitution, compounded by a convoluted seafood supply chain with significant traceability challenges, hinder efforts towards more sustainable, responsible, and ethical fishing and business practices. We conducted the largest evaluation of the quality and accuracy of labels for 672 seafood products sold in Australia, assessing six seafood groups (i.e., hoki, prawns, sharks and rays, snapper, squid and cuttlefish, and tuna) from fishmongers, restaurants, and supermarkets, including domestically caught and imported products. DNA barcoding revealed 11.8% of seafood tested did not match their label with sharks and rays, and snappers, having the highest mislabelling rate. Moreover, only 25.5% of products were labelled at a species-level, while most labels used vague common names or umbrella terms such as ‘flake’ and ‘snapper’. These poor-quality labels had higher rates of mislabelling than species-specific labels and concealed the sale of threatened or overfished taxa, as well as products with lower nutritional quality, reduced economic value, or potential health risks. Our results highlight Australia’s weak seafood labelling regulations and ambiguous non-mandatory naming conventions, which impede consumer choice for accurately represented, sustainable, and responsibly sourced seafood. We recommend strengthening labelling regulations to mitigate seafood mislabelling and substitution, ultimately improving consumer confidence when purchasing seafood.

## Introduction

Global capture fisheries are essential for their contribution to nutrition, food security, and livelihoods^1,2^. Seafood contributes heavily to national economies and is one of the most highly traded foods globally, accounting for 11% of the total agricultural trade (excluding forestry) by value^3^. Yet, the sustainability of fisheries resources has continued to decline globally since the 1970s^4,5^, with almost half of assessed fish stocks overfished and nearly 1 in 10 stocks at the brink of collapse^4–6^. Sustainable fisheries management has focused mostly on fishers and regulators^7^. However, improvements in the seafood supply chain, market-based measures, and consumer influence can positively contribute towards more sustainable, responsible, and ethical fishing and business practices^8–11^.

The seafood supply chain is complex and convoluted, making tracing products from point of harvest through to processing, distribution, and onto the end market a significant challenge^12,13^. The seafood industry is not required to meet the same standards as other commonly consumed products, such as milk and eggs, which have traceability measures in place with mandatory identification and reporting of its country of origin, ingredients, and a unique code, among others^14^. As a result, weak seafood labelling and import regulations create opportunities for substitution of products and can increase the risk of mislabelling and fraud^12,15,16^. This hampers consumer choices for sustainability, undermines sustainable fisheries management by providing an avenue for illegal, unregulated, and unreported (IUU) fishing products to enter legitimate markets, can cause economic loss to governments, and could pose a number of public health and food safety concerns^9,16–20^.

The seafood sector is one of the major food sectors vulnerable to fraudulent activities^12,15,17,20^. Species substitution and seafood mislabelling are the most common forms of seafood fraud and can occur at multiple points in the global supply chain^15,20^. Substitution is predominantly driven by price incentives when a species of lesser value and quality are swapped and sold as more expensive and/or desirable species^21^. It can also occur when a species is marketed as a different product to conceal its origin of harvest if caught in an area closed to fishing, to hide that it was illegally harvested, or to avoid regulation, tariffs, and taxation^11,12,15,20,21^. Mislabelling and substitution could also be driven by a lack of constant supply of a depleting fishery resource to meet market demand and mislead the consumer by concealing the sale of overfished species^16,22,23^. Unintentional mislabelling of seafood is also common, often due to mixed fisheries with similar species being confused and misidentified, or due to weak seafood labelling regulations and ambiguous fish naming standards^11,12,21^.

There is strong interest from Australian seafood consumers for clear and reliable labelling of seafood products (i.e., including the species identity, country of origin and production method)^18,24^. For example, a recent survey by the Marine Stewardship Council found that 9 in 10 Australian seafood consumers want better information on labels to help make sustainable seafood purchases^24^. However, Australia lacks strong labelling regulations and enforcement of standard naming conventions, undermining Australia’s strong fisheries governance framework and robust efforts to prevent overfishing and achieve critical biodiversity targets^25^. Whilst there are standards and regulations in place (i.e., the Australia New Zealand Food Standards Code^14^ and the Country of Origin Food Labelling Information Standard^26^) these are not comprehensive. For example, seafood labelling regulations do not require detailed information on the identity of the species or other key details to trace products from end-to-end, including species scientific name and fishing or farming method^23,27^. Moreover, reporting the country of origin is only required for fresh or frozen seafood sold in Australia, but not for cooked products (except in the Northern Territory). This is concerning given that two thirds of the seafood consumed in Australia is imported^27^, and many of the source countries that Australia imports its seafood from have lower standards of fishing practices or have documented instances of poor fisheries governance and labour practices^6,28,29^.

Naming conventions used for the trade of seafood globally, and on market labels in Australia, are ambiguous, generic, outdated or missing taxa, and often lack the taxonomic resolution required to identify fish to a species level^9,15,30^. The Australian Fish Names Standard AS 5300-2019 (herein AFNS) is a non-mandatory fish naming standard for over 5,000 domestic finfish and an additional 600 commercially important domestic and imported fish species, and allows for the grouping of multiple species with diverse biological traits and geographical origins under single generic ‘umbrella’ labels (e.g., ‘flake’ or ‘snapper’). The AFNS is a voluntary standard with no legal obligation to be enforced (except for the export of Australian seafood products). As a result, different commercial or market names continue to be used for a single species and umbrella terms are misused, extending the number of species referred to under these labels^31^. This compromises the integrity of the seafood market by creating numerous pathways for products to be mislabelled or substituted. Consequently, Australian seafood consumers lack sufficient detail on the seafood products they consume impeding their ability to make informed purchasing decisions.

In more recent years, key seafood markets in the European Union (EU) and the United States (US) have adopted comprehensive legislative frameworks and stringent labelling regulations to ensure seafood traceability and prohibit the importation of threatened or IUU caught species. For example, in 2014 the EU implemented requirements under *Regulation (EU) 1379/2013* for seafood labels to report scientific names, harvest or production method, area of catch (i.e., FAO area, sub-area or division), country of origin, and fishing gear used, among others^32^. The US Food and Drug Administration (FDA) released a Seafood List outlining acceptable common names, market names, and scientific names that are mandated when commercially traded^33^. Additionally, the US FDA adopted DNA based methods for regulatory use to authenticate fish products and monitor seafood mislabelling, ensuring compliance in the seafood market and food safety^34^.

Monitoring the integrity of the seafood market and identifying seafood mislabelling is challenging given that most products are sold already processed for consumption, meaning that morphological features used for species identification are removed^15^. In the absence of morphological identification, DNA barcoding methods are commonly used to accurately and reliably verify the authenticity of seafood and their labels via the sequencing of species-specific gene regions, i.e., a species’ DNA barcode^35–38^. There has been extensive effort to quantify seafood mislabelling, with global rates at the product level estimated between 8 and 25% on average^16,20^. However, some seafood groups are reported to have especially high rates of mislabelling and sampling efforts are highly skewed towards certain taxa and geographies^16,17,20,39^. For example, efforts to evaluate mislabelling are higher in the US, Italy, and Spain, and some of the most common family groups sampled in mislabelling studies include: cods and haddocks; mackerels, tunas and bonitos; sharks; and salmonids^16,31,40^. Consequently, the extent of mislabelling remains largely unknown for many taxa and countries.

In Australia, there are few studies assessing seafood mislabelling, but these are limited in scope and capacity^9,30,31,41^. A study in Tasmania in 2015 assessed 38 seafood samples and while no mislabelling was detected, it recognised naming discrepancies and ambiguity that may cause confusion to consumers^30^. Similarly, a study based in Sydney in 2019 assessed 68 samples and found only 7% were mislabelled, but 40% of fish names used in the labels did not comply with AFNS recommendations^41^. Cawthorn et al. reported 16% of snapper products sold in two states in Australia (32 samples) were mislabelled^9^. And recently, Sharrad et al. reported seafood mislabelling rates for shark products sold in 104 retailers in South Australia, where only 11% of samples were correctly identified, 20% had instances of mislabelling, and the remaining products were ambiguously labelled^31^. While these studies align with international findings that indicate instances of mislabelling^40,42^, there has not been a country-wide assessment of mislabelling across seafood products sold in Australia.

To fill this knowledge gap, we conducted this study that presents the largest effort to date to assess the quality of seafood labelling and rates of seafood mislabelling in Australia. We assessed 672 seafood samples across 6 groups (i.e., hoki, prawns, sharks and rays, snapper, squid and cuttlefish, and tuna), including nationally caught (herein domestic) and imported seafood products sold in Australia. Seafood was purchased at the consumer-end from fishmongers (i.e., a market-style retailer that specifically sells fish), restaurants, and supermarkets across seven of the eight Australian states and territories. Specifically, we addressed three questions: (1) What is the level of taxonomic specificity of seafood labels? (2) Are seafood vendors adopting labels that align with the AFNS? and, (3) what are the mislabelling rates of seafood sold in Australia and how do these vary across seafood products, origin, and outlet type?

## Results

### Label specificity

**Figure 1:**
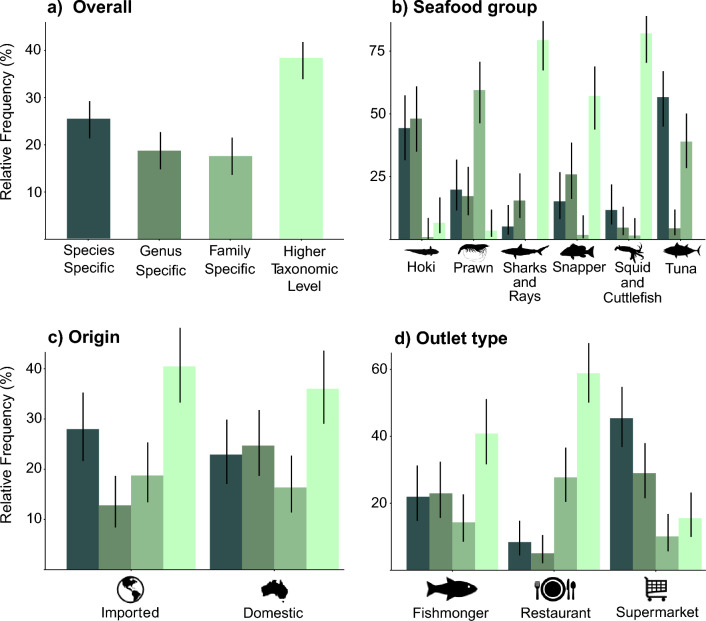
Label specificity frequency distributions (**a**) overall, (**b**) by seafood group, (**c**) by product origin product and (**d**) by outlet type. Colours used refer to the specificity level as defined in the first panel (**a**). Higher taxonomic level refers to labels that include species across multiple families (e.g., shark), or to a non-taxonomic generic name (e.g., fish or fillet). Percentages refer to the proportion of samples under each specificity level for each factor assessed. Error bars represent 95% confidence intervals estimated through the Goodman method.

Label specificity was assessed for all 672 seafood samples collected across Australia (Supplementary Table S2). A quarter of the samples were described in the label at the species level (25.5%) and more than half were described at the family or higher taxonomic level (17.6% and 38.2%, respectively; Fig. 1a), while the remaining 18.8% were labelled at genus level. The averaged ordinal GAM fitted on label specificity included the terms in order of the normalized sum of weights, sustainability certification (1.0), outlet type (1.0), packaging (1.0), seafood group (1.0), Australian state of purchase (1.0), wild caught or aquaculture sourced (0.95, fresh or frozen (0.77), origin (0.52), price per kilogram (0.28). The four models included in the average had an explained deviance ranging between 33.7 and 34.1%.

Label specificity differed significantly between some seafood groups with squid and cuttlefish being the most likely to be labelled less specifically than all other seafood groups relative to the intercept (Est = 4.65, Std Err = 0.38, p =  < 0.001; Fig. 1b; Supplementary Table S4); 80.5% of the squid and cuttlefish samples were labelled at a taxonomic level higher than family. Similarly, the majority of snapper, and shark and ray samples were labelled at a taxonomic level higher than family (57.1% and 79.4%, respectively) and nearly two thirds of prawn samples (59.5%) were labelled at the family level. The seafood groups with the most specific labelling of its products were tuna and hoki, with more than half (56.6%) of tuna products labelled at species level, and almost all (92.5%) of the hoki products labelled at either species or genus level. There was no statistical difference between specificity for tuna and hoki determined from the ordinal GAM (p = 0.07), both as likely as the other to be labelled at a higher specificity than the other four seafood groups (Supplementary Table S4).

Whilst seafood origin added to the explanatory power of the model based on AIC and thus was included in the final averaged GAM, the estimate for the term was not statistically significant (Est = − 0.19, Std Err = 0.25, p = 0.46). Imported products had a bimodal distribution when classified across specificity levels, suggesting that while many products enter the country with species-specific information, many imported seafood products lack taxonomic resolution (Fig. 1c). Products from supermarkets had the most specific labels of the outlet types sampled with nearly half of products labelled at the species level (45.4%). While most products from restaurants (58.8%) and nearly half of products from fishmongers (40.8%) were labelled at a taxonomic level higher than family (Fig. 1d). The specificity of labels from each of the three outlet types were found by the ordinal GAM to be significantly different from each other. Products from supermarkets were predicted to have a higher likelihood of being labelled more specifically than products from a fishmonger (Est = − 0.80, Std. Err = 0.26, p < 0.01), whilst restaurants were more likely to be labelled at a lower specificity than a fishmonger product (Est = 1.31, Std. Err = 0.25, p < 0.001) (Supplementary Table S4).

### Misnaming

Overall, 15.9% of the samples in our study were misnamed. It is important to note that this does not imply the names do not correspond to the species sold, but instead that the label does not use the official nomenclature given in the AFNS. The seafood groups with the highest rates of misnaming were: squid and cuttlefish (32.8%) driven by labels commonly using the term ‘calamari’ that is not recognised on its own by AFNS, snapper (25%) driven by the use of terms such as ‘red snapper’ or ‘pink snapper’, and sharks and rays (20.6%) driven by the use of the undescriptive term ‘shark’ that is not included in AFNS.

### Seafood mislabelling rates

We were able to evaluate mislabelling for 587 products out of the 672 original samples. There were 66 samples removed due to unsuccessful COI barcode amplification, low quality sequence chromatograms, no significant hits in the BOLD and NCBI databases, or only low confidence in hits (where the hit represented the single entry for that species in the sequence database). The 606 sequences retrieved were all > 98% similarity to reference species available in the BOLD and NCBI databases. A further 19 samples were removed due to suspected contamination, because the sample was derived from a mixed animal product and the significant sequence hits were to other animals known to be within the product (e.g., pig from a pork and prawn dumpling).

**Figure 2:**
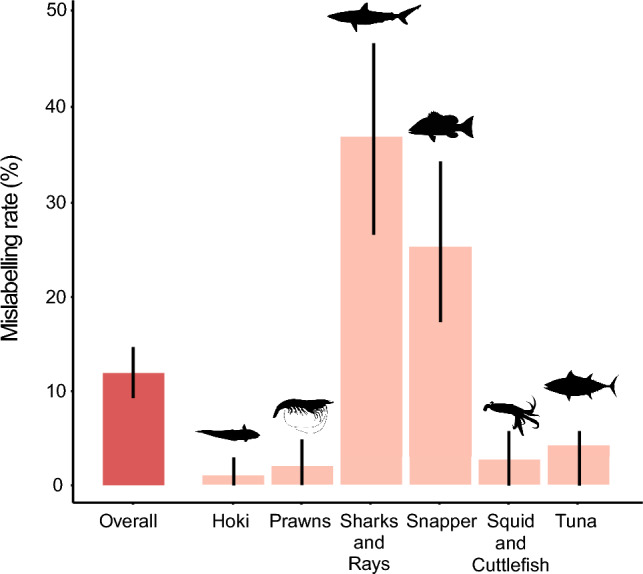
Seafood mislabelling rates overall and by seafood group in Australia as the percentage of labels that did not match the species identified by the DNA genomic analysis. Error bars represent Wald-type 95% confidence intervals. Note that labels included common names and umbrella terms at taxonomic levels higher than species, thus mislabelling occurred when the species identified using DNA barcoding was not within the group of species inferred by the label.

The overall extent of seafood mislabelling at the consumer end in Australia was estimated at 11.8% with labels from 69 products out of 587 samples not matching the species identified in the DNA analysis (Fig. 2). Mislabelling rates varied across levels of label specificity, only 5.0% of samples at a species level were mislabelled, while a greater extent of mislabelling (21.7%) occurred in samples labelled at higher taxonomic level than family (Table 1). While mislabelling was assessed from maximum level of detail in writing only, verbal validation by the vendor was prompted, when possible. However, of the 139 sequenced products that had vendor validation, nearly half (45.3%) were proven to be incorrect identifications of the product. In 4 instances only, the vendor information reverted a product from mislabelled to correct.

**Table 1:** Percentage of seafood samples that are mislabeled overall, by specificity level and based on their origin and outlet type.

	Overall	Origin		Outlet		
		Domestic	Imported	Fishmonger	Restaurant	Supermarket
Sample size	587	306	281	177	213	197
All samples	11.8%	9.2%	14.6%	11.3%	18.8%	4.6%
Specificity level						
Species	5.0%	4.2%	5.8%	4.7%	5.3%	5.1%
Genus	7.7%	5.2%	12.5%	17.1%	10.0%	1.5%
Family	2.1%	4.4%	0.0%	4.0%	1.9%	0.0%
Higher taxonomic level	21.7%	17.1%	25.8%	14.7%	28.0%	11.4%

**Figure 3:**
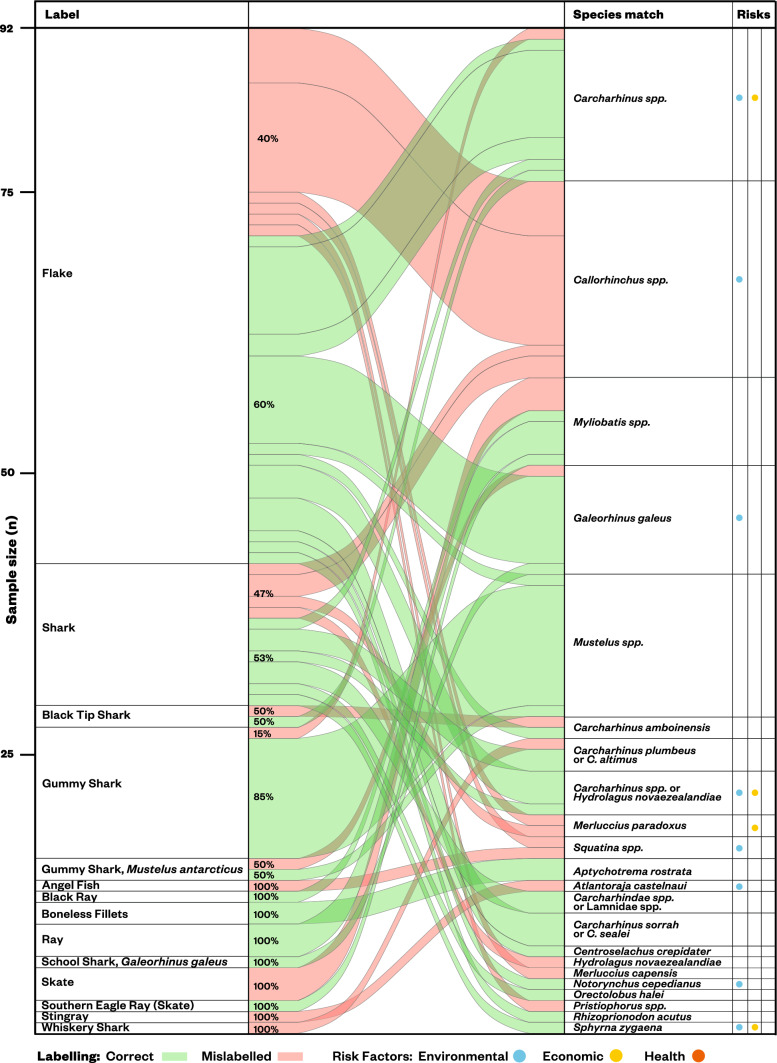
Label to species links for shark and ray products sampled. Risk factors indicate when substitutions can have environmental (i.e., species listed in IUCN Red List or known overfished stocks), economic (e.g., the species identified is of less value than the labelled product), or health (i.e., species with associated consumption risks such as biotoxins) implications to the consumer. Percentage values indicate the proportion of each label category that were correct (green) or mislabelled (red). Reference from scientific to common names used in this study can be found in Table S7.

**Figure 4:**
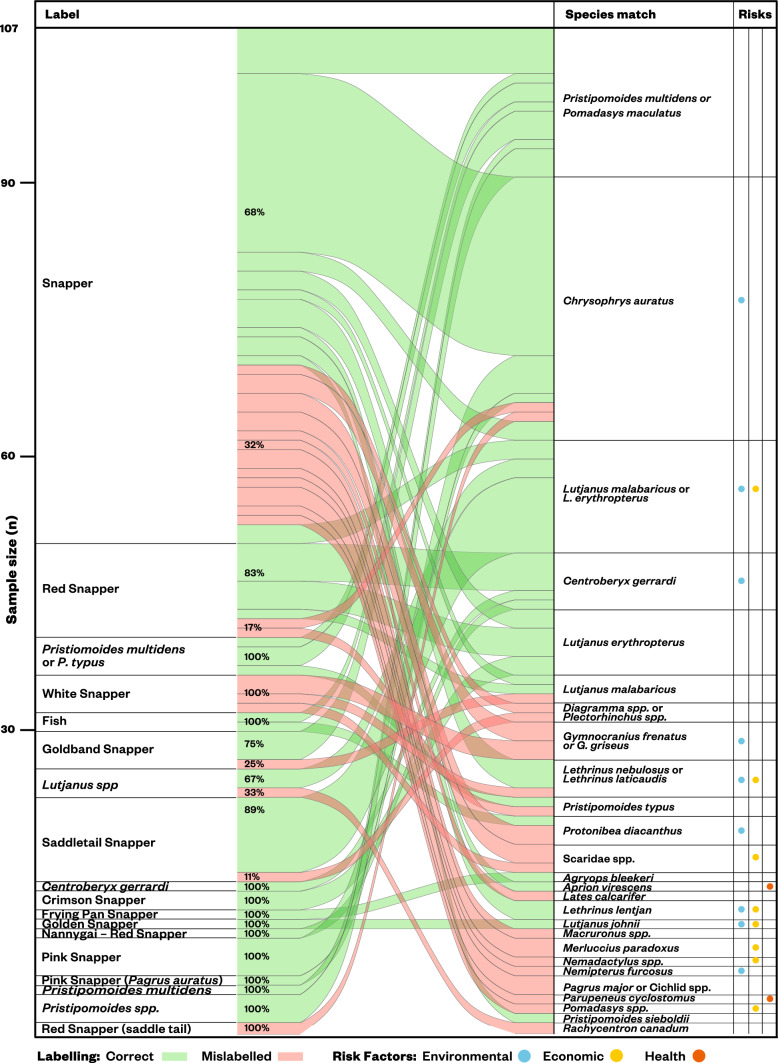
Label to species links identified for snapper products. Risk factors indicate when substitutions can have environmental (i.e., species listed in IUCN Red List or known overfished stocks), economic (e.g., the species identified is of less value than the labelled product), or health (i.e., species with associated consumption risks such as biotoxins) implications to the consumer. Percentage values indicate the proportion of each label category that were correct (green) or mislabelled (red). Reference from scientific to common names used in this study can be found in Table S7.

The seafood group with the highest rate of mislabelling were sharks and rays (35.9%, Figs. 2 and 3) followed by snappers (25.2%, Figs. 2 and 4) then the squid and cuttlefish (12.7%; Fig. 2). Tuna, prawns and hoki were labelled correctly more often than the other three seafood groups, with mislabelling rates at 4.2%, 2.0%, and 1.0%, respectively (Fig. 2). Hoki only had a single case of mislabelling that occurred in a sample labelled at species level. Snapper and sharks and rays had high mislabelling rates in samples labelled least specifically, 34.4% and 39.7% respectively, that is at the taxonomic level higher than family (Supplementary Table S3).

#### Sensitivity analysis of label-to-species definitions

Mislabelling rates varied greatly depending on the label-to-species definition used (see Methods section for details and examples of the definitions used in this study). Had the stricter AFNS definitions of common and umbrella terms been used, rates of mislabelling would have been almost three times higher (34.9%) than those from the more comprehensive definitions used in this study. This change would have important impacts across all seafood groups (Table 2). While the ranking of mislabelling per seafood group remains the same, the AFNS label-to-species definitions would increase rates for all seafood groups. For example, the mislabelling rate for prawns would increase nearly sixfold from 2.0 to 11.9%, and rates for squid and cuttlefish, snapper, and sharks and rays would be 40.7%, 56.1% and 78.3%, respectively. Similarly, using the stricter AFNS definition only for products labelled as ‘flake’, with all other definitions remaining as defined in this study, we would see an increase from 11.8 to 16.7% in the overall mislabelling rate, and from 37.0% to 67.4% when only considering the samples in the shark and rays group. Conversely, if the definition of ‘flake’ was more lenient by including holocephalans, the overall mislabelling rate would decrease to 9.4% overall and it would nearly halve to 20.7% for shark and ray samples.

**Table 2:** Mislabelling rates under different labeling leniencies.

Label-to-species definition	Overall (%)	Hoki (%)	Prawns (%)	Sharks and Rays (%)	Snapper (%)	Squid and cuttlefish (%)	Tuna (%)
In this study	11.8	1.0	2.0	35.9	25.2	12.65	4.2
AFNS definition	34.9	2.9	11.9	78.3	56.1	40.7	16.7
Strict Flake definition	16.7	–	–	67.4	–	–	–
Lenient Flake definition	9.2	–	–	19.6	–	–	–

As per the AFNS definition, strict flake is defined as only referring to the species, *Mustelus antarcticus* and *Mustelus lenticulatus*. Lenient flake definition includes all selachimorphs (sharks) and holocephalans (chimaeras)

–, Infers no change to the mislabelling rate of that seafood group.

#### Drivers of mislabelling

The averaged survival analysis fitted on mislabelling with respect to specificity included the terms (in order of sum of weights): outlet type (1.0), seafood group (1.0), Australian state of purchase (1.0), price per kilogram (0.81), origin (0.80), wild caught or aquaculture sourced (0.49), sustainability certification (0.24), fresh or frozen (0.16).

The model-averaged fit for the survival analysis found significant differences (p < 0.05) in mislabelling rates between seafood groups where hoki, prawn, and tuna had all significantly lower rates than snapper and sharks and rays. Squid and cuttlefish had statistically higher rates of mislabelling than hoki (Est = − 1.1, Std Err = 0.50, p = 0.03), but statistically lower rates than both snapper (Est = − 1.95, Std Err = 0.45, p =  < 0.0001), and shark and rays with the latter found to have the highest rates of mislabelling across the seafood groups sampled (Est = − 2.25, Std Err = 0.45, p =  < 0.0001) (Supplementary Table S5).

There were more instances of mislabelling in imported products (14.6%) than in those of domestic origin (9.2%; Table 1) and, while the AIC model selection process indicates that seafood origin is important in explaining mislabelling, the term for seafood origin was not statistically significant at the p = 0.05 level (Est = 2.3, Std Err = 0.17, p = 0.17). Mislabelling rates varied among different outlet types with fishmongers having a mislabelling rate of 11.3%, this level acted as the intercept for the survival analysis. Restaurants had the highest rates at 18.8%, differing significantly from fishmongers (Est = − 0.66, Std Err = 0.18, p < 0.001), while supermarkets had the lowest rate at 4.6% but were not significantly different from fishmongers (Est = 0.34, Std Err = 0.19, p = 0.06). In particular, the highest rates of mislabelling (28.0%) were found at restaurants in products labelled at a higher taxonomic level than family, although we found no additional effect of an interaction upon exploration (Supplementary Table S5).

#### Standardising seafood mislabelling rates

**Figure 5:**
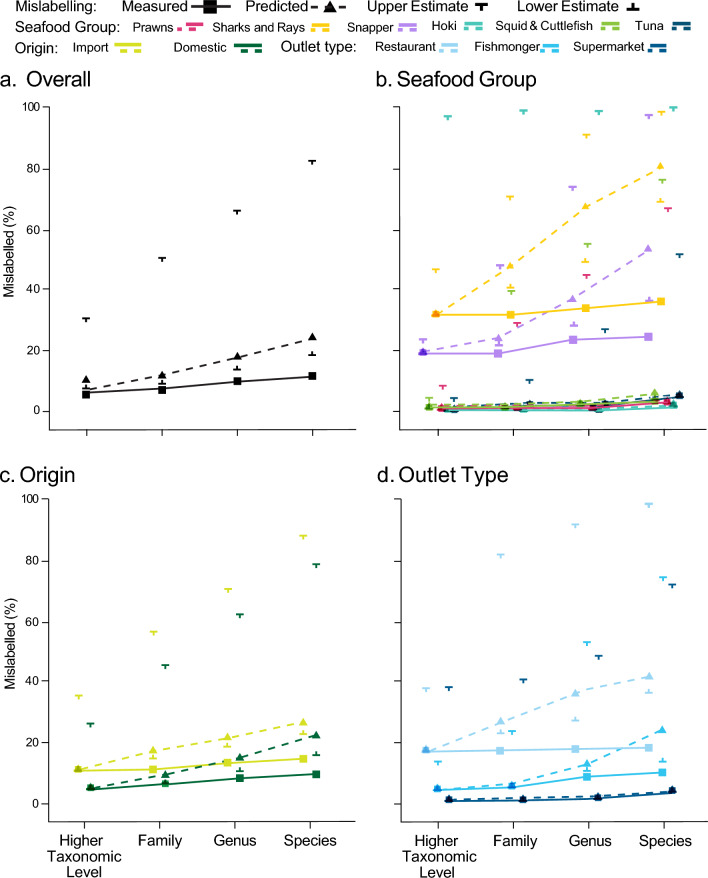
Cumulative incidence curves for censored and predicted mislabelling rates of seafood products in Australia including (**a**) overall, (**b**) by seafood group, (**c**) by product origin and (**d**) by outlet type. Upper and lower estimates represent the bounds of the 95% confidence interval of the predicted mislabelling rate at each specificity level.

Predicting mislabelling rates, assuming all products had been assessed at the species level, we estimated a mislabelling rate of 24.5% overall (95% CI, 19.2–83.3), in comparison with an empirical observation of 11.8% without controlling for censoring (Fig. 5a). By seafood group, sharks and rays had the largest increase in predicted mislabelling when controlling for censoring, with rates doubling to 80.4% (95% CI, 69.6–98.9) if assessed at species level. Similarly, the mislabelling rate for snapper is expected to increase to 54.2% (95% CI, 37.4–98.1), while mislabelling rates for squid and cuttlefish, as well as tuna products, were predicted to increase to 5.3% (95% CI, 2.7–76.1) and 4.2% (95% CI, 4.2–52.8) respectively if assessed at the species level. Hoki and prawns, however, were not predicted to have different mislabelling rates if specificity was standardised to species level and remained at 1.0% (95% CI, 1.0–100.0) and 2.0% (95% CI, 2.0–66.3), respectively (Fig. 5b).

Accounting for censoring, predicted mislabelling rates across seafood origins increased from an observed 14.6% to a predicted 26.7% (95% CI, 22.8–88.3) for imported products, and from 9.2 to 22.5% (95% CI, 16.0–78.8) for domestic (Fig. 5c). And, when all other variables were standardised at their median or most common value, the mislabelling rate was still higher for imported than for domestic seafood products. In regard to outlet types, restaurant products had the greatest departure in mislabelling rates between censored and predicted, from 18.8 to 42.7%, (95% CI, 37.1–100.0) and fishmonger products were predicted to increase from 11.3 to 24.3% (95% CI, 14.1–74.6), if all samples were assessed at species level. Supermarket products were predicted to have a slight increase relative to the censored results from 4.6 to 5.1% (95% CI, 4.6–73.1) (Fig. 5d).

## Discussion

Seafood label quality and mislabelling rates hamper consumer choices for sustainability in Australia due to poor taxonomic resolution, the use of ambiguous umbrella terms and product substitution or misidentification. In the largest evaluation of the quality and accuracy of seafood labels in Australia, we observed that over 1 in 10 seafood products tested did not match their label, and close to three quarters of seafood labels assessed lacked sufficient taxonomic detail for consumers to be informed of what species they are purchasing. We identified that products sold under poor-quality labels, using vague common names or umbrella terms, have higher rates of mislabelling than species-specific labels. For instance, we identified 21.7% mislabelling in products labelled at taxonomic level higher than family, while only 5.0% for products labelled to species level. Our findings align with prior studies, revealing significant differences in label quality and mislabelling rates among seafood groups^16,17^ and outlet types^37,43,44^. Sharks and rays, and snappers, were the seafood groups that had the highest incidence of mislabelling. And products sold in restaurants had less specific labels and higher mislabelling rates than seafood sold at fishmongers or supermarkets.

The reported mislabelling rate of 11.8% is likely a conservative estimate. Had stricter label-to-species definitions as per AFNS been applied here, 34.9% of seafood products would have been considered mislabelled. However, for the purpose of this study, a more comprehensive definition was preferred given that AFNS are not currently mandatory or enforced. Current lenient seafood labelling regulations and vague naming conventions facilitate the use of generic names that group multiple species from different taxonomic groups or with diverse biological traits under umbrella terms, ultimately increasing the risk of mislabelled or incorrectly named products being sold to consumers^9,45^. This is shown in our results as products with species-level identification of the product available in writing to the consumer were less likely to be mislabelled. For example, market names for shark and ray products consistently used ambiguous and often misleading umbrella terms such as ‘flake’ and ‘shark’, which could refer to 326 unique species under our comprehensive definition^46–50^—only 5 of the 97 products sampled had labels at a species-specific level. The use of generic umbrella terms for the sale of shark products to the consumer has been shown to ultimately conceal and misrepresent the identity of the species sold.

Shark and ray products had the highest occurrence of mislabelling of the seafood groups assessed (35.9%). The DNA barcoding analyses revealed that in 18 instances products incorrectly labelled as ‘flake’, or to a lesser extent as ‘shark’, were in fact holocephalans (i.e., Chimaeras) such as C*allorhinchus capensis, Callorhinchus milii,* and *Callorhinchus callorhinchus* (Fig. 3). The high mislabelling rates in shark products found in this study align with previous findings e.g., 20% mislabelled in Australia^31^, or 45% mislabelled in Italy^40^, or 55% mislabelled in Brazil^42^. This is of concern as one third of chondrichthyan species are listed on the IUCN Red List as threatened^51^. Consumers would not be able to confidently identify when they are purchasing a sustainable product or a threatened species. For example, our study recorded two mislabelling cases for Critically Endangered species on the IUCN Red List^52,53^, these were a spotback skate (*Atlantoraja castelnaui*) mislabelled as ‘stingray’, and a school shark (*Galeorhinus galeus*) mislabelled as a ‘gummy shark’—the latter potentially misidentified given these two species are caught in the same fishery^52^. Similarly, pigeye shark (*Carcharhinus amboinensis*), listed as Vulnerable^54^, was sold under the label of ‘flake’, as well as, mislabelled as ‘black tip shark’. Concealed under the label ‘shark’, we identified the sale of 5 taxa that are threatened or of conservation concern including a sandbar shark (*Carcharhinus plumbeus*) listed as Endangered^55^, and a smooth hammerhead (*Sphyrna zygaena*) which may become threatened with extinction without close control of its trade according to Convention on International Trade in Endangered Species of Wild Fauna and Flora (CITES) and is listed as Vulnerable^56^. See Table S7 in Supplementary Materials for a full list of species matched to samples and their IUCN listing.

In Australia, shark meat is a popular seafood often sold under the term ‘flake’, which represented 51.5% of our shark and ray samples. As defined in this study, flake is a catch-all consumer-facing market name used to sell any shark meat^31^. However, according to the AFNS, ‘flake’ is the approved term only for the flesh of gummy shark (*Mustelus antarcticus*) and the New Zealand rig shark (*Mustelus lenticulatus*)—while this definition was approved in 2014, it has not been enforced^50^. The use of this stricter flake definition in our study would have resulted in higher mislabelling rates overall (16.7%) and for the shark and ray group (67.4%). Specific examples of species hidden within the term ‘flake’ included eight incidences of the Critically Endangered school shark (*Galeorhinus galeus*) and a broadnose sevengill shark (*Notorynchus cepedianus*), listed as Vulnerable (Fig. 4).

Snapper products are also labelled using generic and ambiguous terms in Australia and were found to be most likely labelled at a taxonomic level higher than family. A ‘true’ snapper is generally considered to be from the Lutjanidae family^57^, but the use of this market name varies across countries and regions^9^. Our label-to-species definition of the umbrella term ‘snapper’ was comprehensive and, for this study, included 123 unique species from 5 different families (i.e., Lutjanidae, Berycidae, Sparidae, Lethrinidae, and Cheilodactylidae). Yet, snapper had the highest species diversity for mislabelled products, often substituted by species from family groups outside of what is considered a ‘snapper’. Some of the substitutions included tilapia (*Oreochromis spp.*)*,* which has some species listed on the IUCN Red List as Vulnerable^58^, and barramundi (*Lates calcarifer*), a peculiar mislabelling occurrence given this product is generally of higher price^59^ (Fig. 4). The wide diversity of fish names that fall under this umbrella term is problematic for fisheries statistics and traceability given most snapper fisheries are data deficient and poorly managed, and the taxa combined vary considerably in vulnerability and sustainability of their fisheries^9,10^.

There were a number of products labelled as ‘snapper’ that were substituted at some point in the supply chain for lower quality or value species, such as black jewfish (*Protonibea diacanthus*), goldsaddle goatfish (*Parupeneus cyclostomus*), or parrotfish species (Scaridae spp.), suggesting economic incentives behind these substitutions (Fig. 4). Similar substitutions for lower value products were also detected in prawn and tuna products. For example, a product labelled as ‘tiger prawn’ was identified as the lower value ‘vannamei prawn’ (*Penaeus vannamei*), and a tuna product labelled to species-level with ‘*Katsuwonus pelamis’* on its packaging was genetically identified as Alaskan pollock^59^. We also recorded some substitutions of high value products labelled as lower value species, which could indicate an intention to conceal regulatory or environmental concerns of the product being sold, a way to maintain perceived availability of a known product in case of supply issues, or simply an unintentional misidentification^16,21–23^. Examples included products genetically identified as pink snapper (*Chrysophrys auratus*) that were labelled as products of lower quality or value, such as ‘red snapper’ and ‘saddletail snapper’ (Fig. 4). Similarly, a tuna product labelled as a ‘skipjack tuna’ (*Katsuwonus pelamis*) was substituted with a more valuable tuna (Thunnus spp.), this substitution adds potential health risks from inadvertent consumption of higher trophic level species with greater heavy metal content^12,60^.

In considering the label quality and accuracy of products from different outlet types, we found supermarket products to have the most specific labels and the lowest occurrence of mislabelling. Seafood from supermarkets is generally packaged, which can facilitate more detailed information, such as including species names in the ingredients section. This was especially apparent in tuna products, with most supermarket tuna products (88.9%) having species-specific common or scientific names provided on labels, for instance in tuna cans. Conversely, restaurants had the highest mislabelling rates and the lowest specificity of product labels, as most menus provided only family level information like ‘tuna sashimi’ or ‘tuna sushi roll’. Moreover, we found that verbal validation by seafood vendors, mostly possible at restaurants and fishmongers, should not be used as an alternative to written labelling. Vendor identification attempting to improve label specificity did not match our DNA analysis nearly half of the times (45.3%), and only in four instances the vendor validation would have shifted a product from being mislabelled to correct. Thus, reinforcing the need for more specific and accurate written labels^37,43,44^.

Given the high volume of imported seafood consumed in Australia, we assessed labelling of both domestically sourced and imported seafood products. Our results revealed labelling issues at the consumer-end across both origins as very few products, 22.9% of domestic and 28.0% of imported, had sufficient detail to identify the product at a species level. Mislabelling rates were higher for imported at 14.6% than for domestic seafood products at 9.2%. Therefore, the existing framework for seafood import control is insufficient and should capture more details at the border^27^, and strengthening seafood labelling regulations in Australia, for both imported and domestic products, is necessary to improve labelling quality and accuracy at the consumer-end.

DNA barcoding using the mitochondrial COI gene is an established method for species level identification of unknown samples and has proven to be a useful tool to identify seafood mislabelling^15,17,19,30,37,61^. However, the marker may contain insufficient information to discern very closely related taxa, such as members of the genus *Thunnus*^62,63^. This limitation may have contributed to the lower mislabelling rate that we found in tuna products compared to other seafood groups in this study. Furthermore, DNA barcoding technology cannot determine the geographical origin of a fish species—which prevented confirmation of the origin reported in the label, nor the point in the supply chain where mislabelling or substitution occurred, or if it was deliberate or accidental^11,15^. This limitation to identify the stage and intent was further constrained in this study by only sampling products at the consumer end of the seafood supply chain, future efforts could assess mislabelling rates at various stages, including at the border for imported products.

This study offers a novel perspective on seafood mislabelling assessments because we have included a broad range of seafood products sold to consumers, regardless of the level of specificity in the label, rather than limiting our sample to products labelled at species level^9,11,64^. While this approach is a closer representation of the mislabelling experienced by seafood consumers in Australia, we are aware that measuring mislabelling at different levels of specificity could challenge comparison across studies. Therefore, we used a predictive model to control for censoring and estimate mislabelling rates as if all products were assessed at species level. Our findings reveal that seafood mislabelling in Australia is likely closer to 24.5%, which aligns more closely with the upper estimate of the global average range 8–25%^16,20^. Moreover, exploring mislabelling across diverse levels of label specificity showed that consumers target species-specific identification for some products, which may be a market feature (e.g., bluefin tuna that implies certain taste and texture), but demand less specificity for other products where the demand is for any species that meet certain features (e.g., firm mild-flavored white flesh, like flake).

Overall, our results highlight the need for regulatory action to improve traceability in both the domestic and imported seafood supply chains. We recommend improvements to seafood labelling regulations that enforce the use of species-specific common and scientific names by establishing mandatory official fish naming standards that are comprehensive and harmonized among trading countries. Internationally, Harmonised System (HS) trade codes are already established international commodity codes that are implemented to better identify products throughout the supply chain^10^. This system can increase traceability of products, for instance, tuna products already require HS codes and were found to be among the most specifically and accurately labelled when compared to other seafood groups. However, HS trade codes have insufficient taxonomic granularity and could be improved by standardizing nomenclature at a genus and species level and be expanded to incorporate more species. On a national scale, we recommend a comprehensive review of the AFNS and their mandatory enforcement through stronger labelling regulations. If Australia was to adopt better labelling regulations, similar to other global markets such as the EU and US, consumers could be more confident that their seafood is sustainably and legally caught, safe, and honestly labelled.

Seafood mislabelling is prevalent in international seafood supply chains and our study confirms its extent and magnitude across seafood products sold in Australia. Specifically, we provide key baseline knowledge on the specificity and accuracy of seafood labels, highlighting the differences across seafood groups and outlet types. Our conservative mislabelling rates in Australia (11.8%) fall within the global average estimates (8–25%^16,20^) and range from as low as 1.0% of hoki products mislabelled to as high as 35.9% of shark and ray products mislabelled. Moreover, we shine a light on the most controversial umbrella terms that are used domestically (i.e., shark, flake, and snapper) and some of the implications hidden behind vague labelling (e.g., higher mislabelling rates and trade of endangered species, among others)^65^. We also deploy a novel statistical approach that allows fair comparison of mislabelling rates, even in the presence of differing taxonomic resolution among product labels. Our findings should inform policy changes to improve seafood labelling regulations in Australia, which in turn can positively impact fisheries regulation and management, both nationally and internationally, through increased demand for sustainable and traceable seafood products. As a result, consumers should be able to exercise their right to make informed choices and reduce the environmental, economic and health implications associated with ambiguous or inaccurate seafood labelling.

## Methods

### Sample collection

**Figure 6:**
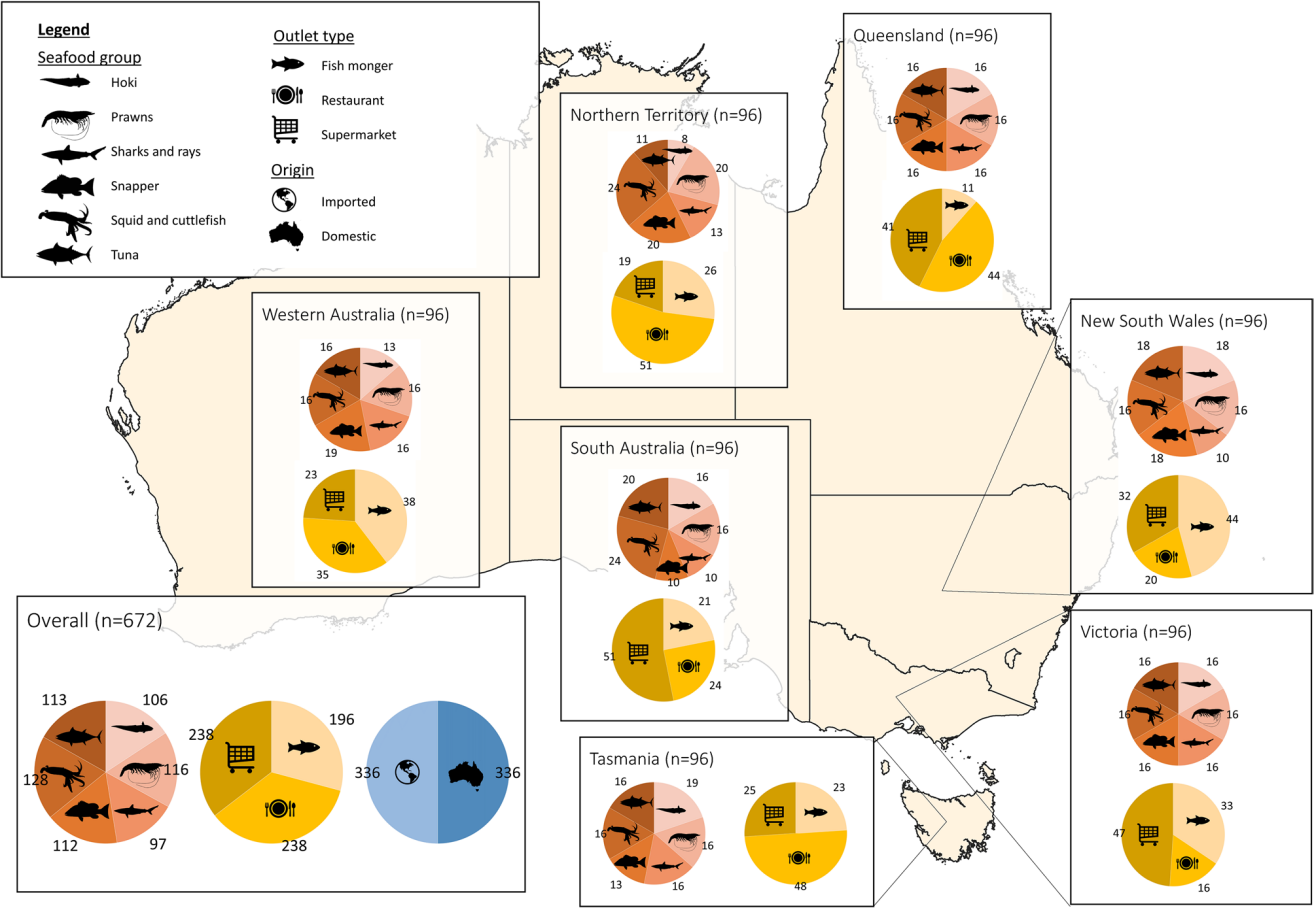
National map of sampling effort. Each state had 96 samples of seafood sampled across 6 different seafood groups (red) and from three types of outlets (yellow). Note the split of origin (blue) was exactly 48:48 in every state thus it is only depicted here for the overall sampling effort.

A total of 672 domestic (n = 336) and imported (n = 336) seafood products were anonymously purchased between May and June 2022 from randomly selected outlets, based on the availability of the seafood of interest. Purchases from fishmongers (n = 196), restaurants (n = 238), and supermarkets (n = 238) were included. To ensure national representation, 96 seafood samples were collected from seven out of eight states and territories in Australia, namely New South Wales, Northern Territory, Queensland, South Australia, Tasmania, Victoria, and Western Australia. Samples were selected across six seafood groups that were identified as either significant species imported into the Australian seafood market or commonly consumed species in Australia^66,67^. These included hoki (n = 106), prawns (n = 116), sharks and rays (n = 97), snapper (n = 112), squid and cuttlefish (n = 128), and tuna (n = 113). The number of samples across location, seafood group, and outlet type were standardised but dependent on the availability of products in each state (Fig. 6). Seafood products were only sampled once per outlet in each state, with the exception of 4 pairs of products where the same product was sampled twice. In these cases, the products were fresh and purchased at a minimum of 1 week apart and were therefore considered independent samples over time.

To provide appropriate representation of the seafood consumed in Australia, the selected seafood products varied including wild caught fisheries, aquaculture farmed, processed (e.g., cooked, frozen, battered, marinated, smoked, peeled, or prepared in a meal) and unprocessed (i.e., raw, fresh), packaged in various forms (e.g., canned or bagged), and ranged from low-value ($3.43/kg) to high-value ($299.90/kg). A summary of the price of seafood products sampled is available in Table S6 and the price of each sample (overall and price per kilogram) are included in the dataset associated with this publication (see data availability statement). Photos of the seafood product and its associated labels, packaging, signage and/or menus were taken at the time of purchase to ensure traceability. In some cases the price per kilogram was unavailable (~ 5.5%), and in these instances we used the R mice package (v3.15.0) to impute the data using a predictive mean matching technique^68^.

Metadata on the product was collected from both the written information available on the label and, when possible, additional details on the product that were provided by the vendor, which were not used for the main assessment but added as additional information. The label information was available either in the packaging, signage, or menu and the following was recorded when available:

1. *Main label* or product name.
2. *Maximum Level of Detail*: the lowest taxonomic identification of the product available for the consumer in writing (i.e., without the need to ask for it). This information may be available in the main label or anywhere else in the associated signs, packaging (e.g., ingredient list), menu or restaurant boards, as long as it is not necessary to ask vendors to access the additional information.
3. *Vendor Validation*: vendors were asked to identify the species of the seafood product if the label was ambiguous (e.g., “market fish”). This was not considered as label information but was recorded separately to assess how this information complements the written details available for the consumer.
4. *Origin*: classification between domestic or imported products. Information on country of origin and country of packing and/or processing was also collected.
5. *Product market data* such as price, price per kilogram, outlet type, third-party certifications (i.e., Marine Stewardship Council, MSC; or Aquaculture Stewardship Council, ASC), processing and packaging information.

### Tissue sampling

Muscle tissue pieces were excised from the seafood product on the same day as purchasing and smeared onto individual Whatman Flinders Technology Associates (FTA) mini, non-indicating cards using sterile equipment as described in Rigby et al.^69^ to purify and preserve nucleic acids for storage at room temperature^70^. Where possible, pieces of muscle tissue were taken from the inner section of the product to avoid contamination of DNA from other species that may be present on the external surface.

### DNA-based specimen identification

All laboratory based genetic methods (DNA extraction, PCR amplification and sequencing) were performed by the Australian Genome Research Facility.

### DNA extraction

DNA extraction was performed on FTA card punches completed using the Qiagen DNeasy Blood and Tissue Kit per the manufacturer instructions (Qiagen, Victoria Australia).

### PCR amplification and sequencing of COI barcode

A portion of the mitochondrial cytochrome c oxidase subunit one (COI) gene region was amplified in a single round of PCR using the forward (m1COlintF (5′–3′) [M13(-12)-F]: [TGTAAAACGACGGCCAGT]GGWACWGGWTGAACWGTWTAYCCYCC) and reverse (jgHCO2198 (5′–3′) [M13-R]: [CAGGAAACAGCTATGACC]TANACYTCNGGRTGNCCRAARAAYCA) primer sequences and assay described by Leray et al.^71^. PCR amplification was performed in 10 μl reaction volumes containing 4 pmol of each primer, 5 µL of Amplitaq Gold 360 2X mastermix and 4 μl of genomic DNA. PCR cycling used a profile of 1 initial cycle of denaturation for 10 min at 95 °C, followed by 35 cycles of denaturation at 94 °C for 1 min, annealing a 47 °C for 2 min and extension at 72 °C for 5 min. The profile was completed with a final extension step at 72 °C for 7 min. Amplification success was confirmed using gel-based QC methods. The amplicon was purified by solid phase reversible immobilisation (SPRI) and labelled using Applied Biosystems™ BigDye™ Terminator v3.1 chemistry. Labelled DNA from the sequencing reaction was again purified then separated and detected on an Applied Biosystems™3730xl Genetic Analyzer.

### DNA barcode sequence analysis and comparison with reference databases

Forward and reverse Sanger sequences were transformed to fastq format using tracy (v0.5.3)^72^. The resulting fastq files were trimmed based on quality scores using fastp (v0.23.2)^73^ with the following parameters: trim quality below 20 (–qualified_quality_phred 20, –cut_mean_quality 20, –average_qual 20), trim 30 bases from the front (–trim_front1 30), trim remaining bases after 250 bp (–max_len1 250), use a cutting window size of 10 bp (–cut_window_size 10) and retain reads longer than 100 bp after trimming (–length_required 100). Reverse reads were reverse-complemented using EMBOSS revseq (v6.6.0.0)^74^.

Each pair of reads was aligned using muscle (v5.1)^75^ with standard settings. The consensus sequence of each pair was generated using a custom script (makeConsensus.py) using the alignment of the two reads and for pairs of disagreeing bases, using the base with the higher quality score. The resulting consensus sequences were compared with NCBI-NT^76^ and BOLD^77^ sequence database (all Arthropoda and Chordata sequences, downloaded 25th August 2022) using blastn (v2.13.0+)^78^ keeping 9,999,999 target sequences per query (–max_target_seqs 9,999,999), filtering by hits with a query coverage of 98%, a base pair identity with BOLD and NCBI databases above 98%, a low e-value (–evalue 1e−10), and assuming distant species hits (-task blastn). BOLD entries with imprecise taxon assignment above the species level (i.e., family- or order-level) were removed from blastn hits.

Both NCBI-NT and BOLD sequence databases contain misidentified or mislabelled entries. To avoid spurious identifications due to such sequence database curation issues, we did not consider matches where the total number of hits was less than 1% of the total entries for that species. We also removed any hits where there was only one blast hit per species in NCBI and BOLD databases to improve confidence in the species assignment. Any hits at taxonomic levels higher than species-level were also omitted (e.g., “*Carcharhinus sp*.” or “unidentified shark fin”).

### Label specificity

Label specificity refers to the maximum level of taxonomic detail available in writing for consumers. All samples were classified based on their label specificity from most specific (i.e., highest precision) to least (i.e., lowest precision) under the following categories:

- *Species level*: label refers to a single species, including a scientific name and/or a common name that can only refer to a single species (e.g., *Thunnus thynnus* or Atlantic Bluefin Tuna)
- *Genus level*: label refers to a genus or to a common name that refers to species within a single genus (e.g., *Mustelus *spp. or Gummy shark)
- *Family level*: label refers to a family or to a common name that refers to species within a single family (e.g., Scombridae or Tuna)
- *Higher taxonomic level*: label refers to a seafood group that includes species across multiple families (e.g., shark), or to a non-taxonomic generic name (e.g., fish or fillet).

In order to define label specificity for each sample, the name recorded as ‘maximum level of detail’ was matched to a reference table of scientific names for all species (and their synonyms) associated with the common names or umbrella terms used in the labels (see Supplementary Table S1). This comprehensive table of label-to-species definitions was compiled for this study using the following databases: World Register of Marine Species^46^, FishBase^47^, the FAO ASFIS List of Species for Fishery Statistics Purposes^49^, Fishes of Australia^48^, and AFNS^50^. The lowest taxonomic level shared between potential species within a given term defined the specificity of the label.

Label specificity was compared across seafood groups, origin, and outlet type to describe patterns in label quality. We treated label specificity as an ordered categorical response and used ordinal regression to determine what factors influence the label specificity associated with each product. The ordinal categorical model was built using a generalised additive approach. We used a model selection process to identify the best models from a candidate set using the R package MuMIn (v1.47.1)^79^. We subsequently averaged the models in the 95% confidence set around the best model to build a model for inference and prediction.

### Misnaming

Samples were considered misnamed when the common name used in the label did not match the AFNS^50^ (available for download at www.fishnames.com.au), which specifies one Standard Fish Name for an individual species or group of species. Misnaming in this context does not imply that the label is incorrect, but instead that it does not use the approved name for the product.

Misnaming was determined by comparing both the maximum level of detail on the product label, along with a simplified version of the main label, with a list of all recognised AFNS terms at either a species or family grouping, or higher grouping level. If the name used in the label for a species did not match the AFNS recommendation, it was considered misnamed. Labels that included a scientific name for the species were considered as named correctly. We used the *amatch* function in the R package *stringdist* (v0.9.10)^80^ to account for slight mismatches such as plurals or spaces when matching against the AFNS labels.

### Mislabelling

In contrast to misnaming, we take mislabelling to be the case when a label on a seafood product does not correspond to the actual product, that is the label is misleading. To evaluate seafood mislabelling, we compared the maximum level of taxonomic detail provided in the original product label to the species identified by DNA barcoding (herein species match). Each sample was classified as correct when the label coincided with the DNA barcode identification, and as mislabelled when they did not. When samples were labelled at higher taxonomic levels than species, these were classified correct when the DNA barcode identification falls within the taxonomic group defined in the label. For example, if a sample was labelled as ‘shark’, it would be considered correct when the DNA barcode identification was any shark (i.e., a species of the subclass Elasmobranchii and superorder Selachimorpha). Alternatively, a sample labelled ‘Gummy shark’, a common name referring to *Mustelus antarticus* and *Mustelus lenticulatus*, was only classified as correct when the DNA barcode identification coincided with either of these species associated to the common name. This step was done by using the same reference table (Supplementary Table S1), as described in specificity section. Results were checked individually to ensure allocations of mislabelling were accurate, including the use of any species synonyms in the label or the DNA sequence databases. For example, king prawn *Penaeus plebejus* has a sequence in the BOLD database labelled under the unaccepted synonym of *Melicertus plebejus,* thus requiring the synonym to be added to the reference table to ensure we do not erroneously identify a mislabeling event.

Mislabelling rates were estimated overall, and compared across seafood groups, origin, and outlet type. Mislabelling rates were also explored for each specificity level to facilitate comparison across studies and because products that are more vaguely labelled have different probabilities to match a species. For example, a product labelled at the species level would only correctly match a single species, while a species labelled at the family level would be considered correct if the match is any species within that family.

#### Sensitivity analysis of label-to-species definitions

Given the potential for variations in market names and species sold under a single common or umbrella term^20^, we conducted a sensitivity analysis to assess the impact of different label-to-species definitions. Our table linking common names and umbrella terms to all possible scientific name matches (Supplementary Table S1) critically informed our decisions on both specificity and mislabeling. For repeatability and transparency, we have therefore published this table alongside our scripts in the repository: https://doi.org/10.5281/zenodo.7962211.

By utilizing multiple sources, we compiled a comprehensive database of species corresponding to the various English names obtained from our sampling. Species can mostly be linked taxonomically to their relevant umbrella terms, for example, terms such as ‘flake’ and ‘shark’ were limited to species within the superorder Selachimorpha. Meanwhile, terms like ‘snapper’ were accepted for taxonomically distinct species where common vernacular names include the word snapper (e.g., Pink snapper). However, these umbrella terms can have a stricter definition under the AFNS—‘flake’ is defined as only two shark species, *Mustelus antarcticus* and *Mustelus lenticulatus* in the AFNS as opposed to our definition that would consider 326 shark species to be a match under this term. Similarly, the term ‘snapper’ refers to 98 species from 2 families in the AFNS but encompasses 123 species from 5 families under our more comprehensive label-to-species definition.

Therefore, we assessed mislabelling rates of Australian seafood products using stricter label-to-species definitions as per the AFNS. We also acknowledge the importance of the umbrella term ‘flake’ in our dataset given its popularity in Australia, its prominence in this study (i.e., 50 flake samples out of 97 shark products) and the ambiguity of this term^31^. We report mislabelling rates of flake products using our comprehensive definition, the stricter AFNS definition (herein ‘strict flake’) and also by including holocephalans such as the commonly caught *Callorhinchus millii* (herein ‘lenient flake’) in our definition. For example, a product labelled as ‘flake’ that was identified as a whaler shark (e.g., *Carcharhinus obsurus*) was considered not mislabelled according to our label-to-species definition or to the lenient flake definition, but would be mislabelled according to the AFNS or strict flake definition.

### Statistical analysis

Studies of mislabelling rates have historically struggled with the problem that different products are labelled at different taxonomic levels. A common solution to this problem has been to limit sampling to products that are labelled at a species level^9,64^. However, this introduces a sampling bias, as those products are likely to differ in important ways from others that are labelled more generically—for instance in price, market demand for information, ease of visual identification, or aspects of consumption.

We treated products labelled above species level as being right-censored measurements—in statistics, right-censoring is a condition in which the value of a measurement is only known at the last point of measurement^81,82^. In our case, measurement was taken in intervals at each of the levels of specificity, analogous to periods of time in traditional survival analyses, with most products not measured at the species level. The *survreg* function in the R package Survival (v3.4.0)^83^ allows for a model to be built with mislabelling as the response variable with respect to specificity as the sampling interval. A survival regression was built using the same predictors as the ordinal regression described above. Again, we used the MuMin package to average the top 95% models resulting in the final model selection.

#### Predictive analysis of seafood mislabelling

To remove label specificity bias and facilitate comparison across studies, we estimated the predicted mislabelling rates if all products sampled had been assessed at the species level. To standardize mislabelling rates at the species level we used the survival analysis model developed above to predict the highest specificity at which the product is expected to be correctly labelled or conversely the specificity at which we expect mislabelling to occur, if it does.

Given any product mislabelled at any higher taxonomic level would also be incorrect at the next most specific level, proportions of mislabelling are cumulative from lower to higher specificity. A cumulative incidence curve across specificity levels was produced to determine the censored (i.e., measured) and predicted mislabelling rates at each level of specificity. The margin of error using the 95% confidence interval around the predicted point of mislabelling for each product were considered to generate an upper and lower estimate of predicted mislabelling rates. These were plotted as error bars around the predicted curve to show the deviation of the model’s results. Moreover, we standardised all variables (i.e., using median or the most common values) and explored specifically the effect of seafood origin to determine if imported products were more likely to be mislabelled.

## Supplementary Information

Supplementary Information 1.

Supplementary Information 2.

Supplementary Information 3.

## Data Availability

The datasets generated and analysed during the current study are available in the Zenodo repository, https://doi.org/10.5281/zenodo.7962211.
