# Supplementary material for: Seafood label quality and mislabelling rates hamper consumer choices for sustainability in Australia
Source: Sci Rep. 2023 Aug 3;13:10146. doi: 10.1038/s41598-023-37066-4 (PMC10400555; doi:10.1038/s41598-023-37066-4)
Supplement: Supplementary file 2 — Supplementary Information 2. [file 41598_2023_37066_MOESM2_ESM.docx]

**Seafood label quality and mislabelling rates hamper consumer choices for sustainability in Australia**

Megan E. Cundy^1^, Alexander G. McLennan^1^, Chris Wilcox^1, 2^, Marcelle E. Ayad^1^, Philipp E. Bayer^1^, Madalyn Cooper^1^, Shannon Corrigan^1^, Emily Harrison^1^ and Julia Santana-Garcon*^,1, 2^

^1^Flourishing Oceans Initiative, Minderoo Foundation, Perth, Western Australia, Australia

^2^Centre for Marine Socioecology, University of Tasmania, Hobart, Tasmania 7001, Australia

*Corresponding author, [jgarcon@minderoo.org](mailto:jgarcon@minderoo.org)

**Supplementary materials**

**Supplementary Table S1.** A compilation of binomial species names associated with each of the maximum level of detail available anywhere on the product's packaging in our study. Taxonomic granularity at which they specify is recorded under specificity. Binomial names were identified as associated with the label through a review of World Register of Marine Species, FishBase, the FAO ASFIS List of Species for Fishery Statistics Purposes, Fishes of Australia, and AFNS.

**See table as excel file.*

**Supplementary Table S2.** Specificity by seafood group shown as the proportion of samples (%) in each seafood group that had labels at each specificity category.

|  | Specificity level | Overall | Hoki | Prawns | Sharks & Rays | Snapper | Squid & Cuttlefish | Tuna |
| --- | --- | --- | --- | --- | --- | --- | --- | --- |
| All samples | *Sample size* | *672* | *106* | *116* | *97* | *112* | *128* | *113* |
|  | Species level | 25.5 % | 44.3 % | 19.8 % | 5.2 % | 15.2 % | 13.3 % | 56.7 % |
|  | Genus level | 18.8 % | 48.1 % | 17.2 % | 15.4 % | 25.9 % | 4.7 % | 4.4 % |
|  | Family level | 17.6 % | 1.0 % | 59.5% | 0.0 % | 1.8 % | 1.5 % | 38.9 % |
|  | Higher taxonomic level | 38.2 % | 6.6 % | 3.5 % | 79.4 % | 57.1 % | 80.5 % | 0.0 % |
| Domestic Samples | *Sample size* | *336* | *52* | *56* | *57* | *60* | *60* | *51* |
|  | Species level | 22.9 % | 46.2 % | 7.1 % | 8.8 % | 25.0 % | 5.0 % | 52.9 % |
|  | Genus level | 24.7 % | 50.0 % | 35.7 % | 19.3 % | 28.3 % | 6.7 % | 9.8 % |
|  | Family level | 16.4 % | 0.0 % | 57.1 % | 0.0 % | 3.3 % | 3.3 % | 37.3 % |
|  | Higher taxonomic level | 36.0 % | 3.8% | 0.0 % | 1.7 % | 43.3 % | 85.0 % | 0.0 % |
| Imported Samples | *Sample size* | *336* | *54* | *60* | *40* | *52* | *68* | *62* |
|  | Species level | 28.0 % | 42.6 % | 31.7 % | 0.0 % | 3.9 % | 20.6 % | 59.7 % |
|  | Genus level | 12.8 % | 46.3 % | 0.0 % | 10.0 % | 23.1 % | 2.9 % | 0.0 % |
|  | Family level | 18.8 % | 1.9 % | 61.7 % | 0.0 % | 0.0 % | 0.0 % | 40.3 % |
|  | Higher taxonomic level | 40.5 % | 9.2 % | 6.7 % | 90.0 % | 73.1 % | 76.5 % | 0.0 % |
| Fishmonger Samples | *Sample size* | *196* | *12* | *28* | *32* | *50* | *41* | *33* |
|  | Species level | 21.9 % | 75.0 % | 7.1 % | 9.4 % | 14.0 % | 4.9 % | 60.6 % |
|  | Genus level | 22.9 % | 16.7 % | 35.7 % | 21.9 % | 36.0 % | 9.7 % | 12.1 % |
|  | Family level | 14.3 % | 8.3 % | 53.6 % | 0.0 % | 2.0 % | 4.9 % | 27.3 % |
|  | Higher taxonomic level | 40.8 % | 0.0 % | 3.6 % | 68.7 % | 48.0 % | 80.5 % | 0.0 % |
| Restaurant Samples | *Sample size* | *238* | *23* | *38* | *54* | *39* | *49* | *35* |
|  | Species level | 8.4 % | 65.2 % | 0.0 % | 0.0 % | 2.6 % | 0.0 % | 11.4 % |
|  | Genus level | 5.1 % | 21.8 % | 5.3 % | 5.6 % | 0.0 % | 2.0 % | 2.9 % |
|  | Family level | 27.7 % | 0.0 % | 94.7 % | 0.0 % | 0.0 % | 0.0 % | 85.7 % |
|  | Higher taxonomic level | 58.8 % | 13.0 % | 0.0 % | 94.4 % | 97.4 % | 98.0 % | 0.0 % |
| Supermarket Samples | *Sample size* | *238* | *71* | *50* | *11* | *23* | *38* | *45* |
|  | Species level | 45.4 % | 32.4 % | 42.0 % | 18.2% | 39.1 % | 39.5 % | 88.9 % |
|  | Genus level | 29.0 % | 62.0 % | 16.0 % | 45.4 % | 47.8 % | 2.6 % | 0.0 % |
|  | Family level | 10.0 % | 0.0 % | 36.0 % | 0.0 % | 4.4 % | 0.0 % | 11.1 % |
|  | Higher taxonomic level | 15.6 % | 5.6 % | 6.0 % | 36.4 % | 8.7 % | 57.9 % | 0.0 % |

**Supplementary Table S3.** Mislabelling by seafood group and specificity level.

| Specificity group | Hoki | Prawns | Sharks & Rays | Snapper | Squid & Cuttlefish | Tuna |
| --- | --- | --- | --- | --- | --- | --- |
| *Sample size* | *102* | *101* | *92* | *107* | *113* | *72* |
| **Overall** | **1.0 %** | **2.0 %** | **35.9 %** | **25.2 %** | **12.7 %** | **4.2 %** |
| **Specificity level** |  |  |  |  |  |  |
| Species level | 2.2 % | 0.0 % | 40.0 % | 5.8 % | 6.7 % | 5.4 % |
| Genus level | 0.0 % | 5.6 % | 14.3 % | 18.5 % | 25 % | 0.0 % |
| Family level | 0.0 % | 1.7 % | NA | 0.0 % | 0.0 % | 3.3 % |
| Higher taxonomic level | 0.0 % | 0.0 % | 39.7 % | 34.4 % | 1.1 % | NA |

**Supplementary Table S4.** Coefficients of the averaged ordinal generalised additive regression model run on specificity of seafood product labelling whereby a high estimate is characterised by low taxonomic resolution and therefore low specificity compared to the intercept.

|  | **Estimate** | | **Std. E** | | **z value** | | **Pr(>\|z\|)** | | **Sig** | |  |  |
| --- | --- | --- | --- | --- | --- | --- | --- | --- | --- | --- | --- | --- |
| (Intercept) | | -2.82 | | 0.69 | | 4.06 | | <0.01 | | *** | |  |
| Certification: unknown | | 0.11 | | 0.35 | | 0.30 | | 0.76 | |  | |  |
| Certification: yes | | 1.77 | | 0.35 | | 5.11 | | <0.01 | | *** | |  |
| Freshness: frozen | | 0.51 | | 0.36 | | 1.39 | | 0.17 | |  | |  |
| Freshness: unknown | | 0.58 | | 0.46 | | 1.25 | | 0.21 | |  | |  |
| Outlet Type: restaurant | | 1.31 | | 0.25 | | 5.29 | | <0.01 | | *** | |  |
| Outlet Type: supermarket | | -0.80 | | 0.26 | | 3.04 | | <0.01 | | ** | |  |
| Packaging: canned | | -0.97 | | 0.55 | | 1.75 | | 0.08 | | . | |  |
| Packaging: no | | 0.65 | | 0.26 | | 2.54 | | 0.01 | | * | |  |
| Seafood Group: prawns | | 1.66 | | 0.32 | | 5.10 | | <0.01 | | *** | |  |
| Seafood Group: sharks | | 4.08 | | 0.40 | | 10.10 | | <0.01 | | *** | |  |
| Seafood Group: snapper | | 2.82 | | 0.36 | | 7.94 | | <0.01 | | *** | |  |
| Seafood Group: squid | | 4.65 | | 0.38 | | 12.16 | | <0.01 | | *** | |  |
| Seafood Group: tuna | | 0.66 | | 0.36 | | 1.84 | | 0.07 | | . | |  |
| State: NT | | -0.17 | | 0.40 | | 0.41 | | 0.68 | |  | |  |
| State: QLD | | 0.06 | | 0.32 | | 0.32 | | 0.18 | | 0.86 | |  |
| State: SA | | -0.82 | | 0.33 | | 0.33 | | 2.51 | | 0.01 | | * |
| State: TAS | | -1.11 | | 0.33 | | 0.33 | | 3.33 | | <0.01 | | *** |
| State: VIC | | -0.43 | | 0.31 | | 0.31 | | 1.38 | | 0.17 | |  |
| State: WA | | 0.24 | | 0.34 | | 0.34 | | 0.70 | | 0.48 | |  |
| Wildcaught or Aquaculture: unknown | | 1.09 | | 0.52 | | 0.52 | | 2.09 | | 0.04 | | * |
| Wildcaught or Aquaculture: wildcaught | | 0.66 | | 0.48 | | 0.48 | | 1.37 | | 0.17 | |  |
| Origin: domestic | | -0.19 | | 0.25 | | 0.26 | | 0.74 | | 0.46 | |  |
| PricePerKg | | <0.01 | | <0.01 | | <0.01 | | 0.30 | | 0.77 | |  |
| one level of each factor is included in the intercept term as the reference level. The reference levels are:  Coastline shape – Concave, Substrate – Boulder, Backshore type – Cliff. Spatial smooth terms are constrained to have a mean of 0, and thus represent deviations from the  parametric terms due to spatial patterns  one level of each factor is included in the intercept term as the reference level. The reference levels are:  Coastline shape – Concave, Substrate – Boulder, Backshore type – Cliff. Spatial smooth terms are constrained to have a mean of 0, and thus represent deviations from the  parametric terms due to spatial patterns  Note that one level of each factor is included in the intercept term as the reference level. The reference levels are: Certification – *No*, Freshness – *Fresh*, Outlet Type – *Fishmonger*, Packaging – *Packaged*, Seafood Group – *Hoki*, State – *NSW*, Wild caught or Aquaculture – *Aquaculture*, Origin – *Import*. | | | | | | | | | | | | |

**Supplementary Table S5.** Coefficients of the averaged survival analysis regression model run on mislabelling of seafood product labelling with specificity as the interval whereby a high estimate is characterised by less likelihood of mislabelling compared to the intercept.

|  | **Estimate** | **Std. Error** | **Z value** | **Pr(>\|z\|)** | **Sig** |
| --- | --- | --- | --- | --- | --- |
| (Intercept) | 3.00 | 0.58 | 5.16 | <0.01 | *** |
| Origin: domestic | 0.23 | 0.17 | 1.38 | 0.17 |  |
| Outlet Type: restaurant | -0.70 | 0.18 | 3.93 | <0.01 | *** |
| Outlet Type: supermarket | 0.34 | 0.19 | 1.86 | 0.06 | . |
| PricePerKg | <0.01 | 0.00 | 1.42 | 0.16 |  |
| Seafood Group: prawns | -0.59 | 0.52 | 1.13 | 0.26 |  |
| Seafood Group: sharks | -2.20 | 0.45 | 4.84 | <0.01 | *** |
| Seafood Group: snapper | -1.94 | 0.45 | 4.33 | <0.01 | *** |
| Seafood Group: squid | -1.08 | 0.49 | 2.20 | 0.03 | * |
| Seafood Group: tuna | -0.79 | 0.49 | 1.60 | 0.11 |  |
| State: NT | 0.30 | 0.26 | 1.17 | 0.24 |  |
| State: QLD | -0.18 | 0.22 | 0.84 | 0.40 |  |
| State: SA | 0.03 | 0.26 | 0.11 | 0.91 |  |
| State: TAS | 0.35 | 0.26 | 1.32 | 0.19 |  |
| State: VIC | 0.46 | 0.28 | 1.65 | 0.10 | . |
| State: WA | -0.25 | 0.22 | 1.12 | 0.26 |  |
| Wildcaught or Aquaculture: unknown | -0.89 | 0.09 | 10.01 | <0.01 | *** |
| Wildcaught or Aquaculture: wildcaught | -0.01 | 0.32 | 0.02 | 0.98 |  |
| Log(scale) | 0.13 | 0.35 | 0.37 | 0.71 |  |
| Certificationunknown | -0.04 | 0.15 | 0.27 | 0.79 |  |
| Certificationyes | -0.14 | 0.30 | 0.46 | 0.65 |  |
| Freshnessfrozen | -0.01 | 0.09 | 0.16 | 0.87 |  |
| Freshnessunknown | -0.03 | 0.13 | 0.24 | 0.81 |  |
| Note that one level of each factor is included in the intercept term as the reference level. The reference levels are: Origin – *Import,* Outlet Type – *Fishmonger*, Seafood Group – *Hoki*, State – *NSW*, Wild caught or Aquaculture – *Aquaculture*, Certification – *No*, Freshness – *Fresh*. | | | | | |
| **Supplementary Table S6 –** Price summary of the seafood groups sampled. Where a specific price per kilogram was absent from the point of sale, an estimate was calculated from the total price of the product divided by the weight. This means that some battered products may have been overestimated.   \| **Seafood group** \| **Mean**  **(AUD)** \| **Std. Dev**  **(AUD)** \| **Min**  **(AUD)** \| **Max**  **(AUD)** \| \| --- \| --- \| --- \| --- \| --- \| \| Hoki \| 27.68 \| 16.28 \| 10.68 \| 119.92 \| \| Prawns \| 47.00 \| 29.83 \| 3.43 \| 160.00 \| \| Sharks and rays \| 44.19 \| 28.27 \| 5.99 \| 130.00 \| \| Snapper \| 47.85 \| 26.69 \| 9.32 \| 220.00 \| \| Squid and cuttlefish \| 32.05 \| 28.47 \| 9.50 \| 181.30 \| \| Tuna \| 51.67 \| 45.30 \| 3.43 \| 299.90 \|   **Supplementary Table S7 –** Genomic matches to each combination of seafood group, state, outlet type, origin, and label including the species IUCN red listing. The final column counts the number of instances that combination of factors matched the exact same genomic output. Contaminated products are not shown.  **See table as excel file.* | | | | | |

one level of each factor is included in the intercept term as the reference level. The reference levels are:

Coastline shape – Concave, Substrate – Boulder, Backshore type – Cliff. Spatial smooth terms are constrained to have a mean of 0, and thus represent deviations from the

parametric terms due to spatial patterns

one level of each factor is included in the intercept term as the reference level. The reference levels are:

Coastline shape – Concave, Substrate – Boulder, Backshore type – Cliff. Spatial smooth terms are constrained to have a mean of 0, and thus represent deviations from the

parametric terms due to spatial patterns
